# On the approximation of sum of lognormal for correlated variates and implementation

**DOI:** 10.1371/journal.pone.0325647

**Published:** 2025-06-23

**Authors:** Asyraf Nadia Mohd Yunus, Nora Muda, Abdul Rahman Othman, Sonia Aïssa

**Affiliations:** 1 Department of Mathematical Sciences, Faculty of Science and Technology, Universiti Kebangsaan Malaysia, UKM Bangi, Selangor, Malaysia; 2 Universiti Sains Malaysia, Pulau Pinang, Malaysia; 3 Institut National de la Recherche Scientifique, Énergie Matériaux Télécommunications, Québec City, Canada; Villanova University, UNITED STATES OF AMERICA

## Abstract

In probabilistic modeling across engineering, finance, and telecommunications, sums of lognormal random variables frequently occur, yet no closed-form expression exists for their distribution. This study systematically evaluates three approximation methods—Wilkinson (W), Schwartz & Yeh (SY), and Inverse (I)—for correlated lognormal variates across varying sample sizes and correlation structures. Using Monte Carlo simulations with 5, 15, 25, and 30 samples and correlation coefficients of 0.3, 0.6, and 0.9, we compared Type I error rates through Anderson-Darling goodness-of-fit tests. Our findings demonstrate that the Wilkinson approximation consistently outperforms the other methods for correlated variates, exhibiting the lowest Type I error rates across all tested scenarios. This contradicts some previous findings in telecommunications literature where SY was preferred. We validated these results using real-world datasets from engineering (fatigue life of ball bearings) and finance (stock price correlations), confirming the Wilkinson approximation’s superior performance through probability density function comparisons. This research provides practical guidance for selecting appropriate approximation methods when modeling correlated lognormal sums in diverse applications.

## Introduction

The lognormal distribution has a long history of use in various fields, particularly in engineering, environmental science, and finance. It is a crucial distribution in statistical analysis, especially when dealing with real-world datasets. Many real-world scenarios involve the sum of lognormal variates, but since no closed-form probability distribution exists for this sum, approximation methods become necessary.

Past research has highlighted the importance of determining the probability density function for a sum of log-normally distributed random variables. For instance, Beaulie et al. [1] explored applications in inventory management for estimating distributions of independent and identically distributed variables. Cardieri & Rappaport [2] identified significant applications in wireless communication, noting that the exact distribution of signals composed of lognormal variates is unknown. Additionally, Cobb et al. [3] developed a process using the Fenton-Wilkinson approximation to estimate parameters for a single lognormal probability distribution function, with particular relevance to stock market time series.

### Lognormal distribution fundamentals

The lognormal distribution is characterized by two primary parameters: mean and standard deviation. When a variable *X* is lognormal (or ln(*X*) is normal), the probability density function can be expressed mathematically [4] as:


$$g\left(x|\mu ,\sigma^{2}\right)=\frac{1}{\sqrt{2\pi }\;\sigma x}\exp\left(-\frac{{\left(\ln(x)-\mu \right)}^{2}}{2\sigma^{2}}\right),\;\;\;\;\;\;\;\;\;\;\;\;x>0.\;$$

Where *x* > 0, *μ* is the mean of the natural logarithm of the variable and *σ* is the standard deviation of the natural logarithm of the variable. Maximum likelihood estimation for *μ* and *σ* can be calculated using specific formulas:



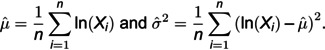



### Challenges in summing lognormal variates

The summation of lognormal distributions presents significant computational challenges due to the lack of a known closed form. Researchers have found that the sum of lognormal variates does not necessarily result in another lognormal variate. Santos Filho, Cardieri & Yacoub [5,6] demonstrated that the sum of lognormal variates can produce different outcomes compared to individual lognormal variates. Furthermore, Othman et al. [7] examined the sum of lognormal variates under various conditions, including independent and identically distributed (IID) and non-identically distributed (NIID) scenarios

### Research gap and contributions

Despite the extensive use of lognormal distributions in various fields, a significant research gap exists in determining which approximation method performs best for the sum of correlated lognormal variates across different sample sizes and correlation structures. While previous works by Cardieri & Rappaport [2] and [5,6] established foundational approaches, they primarily focused on independent variates or specific application contexts without systematically comparing multiple approximation methods across various correlation strengths. This study addresses this gap by examining the specific research question: “Which approximation method—Wilkinson, Schwartz and Yeh, or Inverse—provides the most accurate estimation for the sum of correlated lognormal variates across various sample sizes and correlation strengths?” Our work makes several novel contributions: (1) it provides the first systematic comparison of all three major approximation methods specifically for correlated lognormal variates; (2) it establishes empirical evidence for optimal approximation selection based on sample size and correlation strength; (3) it validates findings using diverse real-world applications from both engineering and finance; and (4) it employs rigorous statistical testing through Type I error rates and Anderson-Darling tests to determine the most reliable approximation method. This comprehensive approach extends beyond previous research that was often limited to specific applications or did not fully explore the interactions between correlation structure and approximation accuracy.

Therefore, based on the previous research, the problem related to the sum of lognormal variate which is not estimated to be equal to the other lognormal variates need to be studied and examined. This study used three methods of approximation which are Wilkinson (W), Schwartz & Yeh (SY) and Inverse Approximation (I). Prior to comparing the three approximations, firstly the result of normal simulation was derived to estimate the sum of lognormal. The Anderson- Darling goodness of fit was then used in this paper as an evaluation test to define the best approximation. Hence the approximations were also applied on real data to state the result of getting the best estimation.

### Understanding sum of lognormal approximation

#### Correlated lognormal variates: theoretical background.

The study of correlated lognormal variates holds significant importance in various fields, ranging from finance and insurance to engineering and environmental sciences. The lognormal distribution, characterized by its ability to capture skewness and heavy-tailed behavior, has been widely employed to model a variety of phenomena, including traffic patterns in the internet and financial risk factors [8]. The performance of the lognormal distribution in predicting traffic volumes in the internet has been demonstrated, outperforming traditional Gaussian and Weibull distributions [8]. The modeling of data breaches using the log-skew-normal distribution has also shown promising results, highlighting the importance of considering skewed distributions in actuarial modeling [9]. The findings from these studies suggest that the lognormal distribution and its variants, such as the log-skew-normal distribution, are well-suited for capturing the characteristics of various correlated phenomena. The theory of correlated lognormal variates has garnered significant attention in the field of statistical modeling, as it provides a versatile framework for analyzing and understanding the behavior of variables that exhibit a lognormal distribution.

A lognormal random variable X is defined as $X=e^{Y}$ where Y is normally distributed. If $Y\sim N\left(\mu ,\sigma^{2}\right)$, then X has a lognormal distribution with mean and variance as follows:


$$E(X)=e^{\mu +\frac{\sigma^{2}}{2}}$$

$$Var(X)=\left(e^{\sigma^{2}}-1\right)e^{2\mu +\sigma^{2}}$$

When dealing with multiple lognormal, the variates may not be independent. In most cases, correlation occurs between the variates when the underlying normal distributions are associated. For the correlated lognormal variates, let $Y=\left[Y_{1},Y_{2},...,Y_{n}\right]$ be a multivariate normal vector with mean vector and covariance matrix. If $X_{i}=e^{Y_{i}}$ for i=1,...,n, then $X=\left[X_{1},X_{2},...,X_{n}\right]$ is a vector of correlated lognormal variates. The structure of the $X_{i}$’s depends on the covariance structure covariance matrix of the normal vector Y. There are two samples set, then the correlation between $X_{i}$ and $X_{j}$ is derived from the covariance matrix of the underlying normal distribution particularly $Corr(X_{i},X_{j})=\frac{e^{\sum ij}-1}{\sqrt{\left(e^{\sum ii}-1\mathrm{\backslash rightleft}(e^{\sum jj}-1\right)}}$ where ∑ij is the covariance between $Y_{i}$ and $Y_{j}$.

The sum of correlated lognormal variates is a complex statistical issue with major uses in wireless communications, risk analysis and finance. When *X* and *Y* are correlated, the complexity increases [10]. While the exact distribution of this sum lacks a closed analytical form, several approximation methods have been developed. The sum of correlated lognormal data is approximated widely and for example by using a long-extended-skew-normal distribution or numerical integration techniques. Even if each lognormal variable has good mathematical characteristics, but the sum of the variables will result an incomprehensible integral that cannot be reduced to simple functions. The correlation structure between the variables adds another element of complexity, as it affects both the mean and variance of the sum in non-linear ways. Beaulieu & Qiong [1] mentioned that the sum of lognormal variates has been applied in numerous real-world problems, especially in wireless systems by modelling the amount of network interference signals in a shadowed propagation environment as a sum of *N* whose signals are lognormally distributed. In other paper, Maoke & Xiaofeng (2022) approximate closed-form probability density function expression for the sum of lognormal-Ricia turbulence channels with Rayleigh pointing errors is developed.

The complexity of summing correlated lognormal variates stems from the inability to derive a closed analytical form. Various approximation methods have been developed to address this challenge, each with its own strengths and limitations. This study aims to provide a comprehensive understanding of these methods and their applicability across different contexts.

A widely used assumption is the sum of distributions is approximated by other lognormal variates, which is


$$W=\sum_{i=1}^{N}X_{i}=X_{1}+X_{2}+X_{3}+...+X_{N}\approx Z$$ (1)

whereby the variable *Z* is lognormally distributed. In this research, the mean value and standard deviation value of the sum of lognormal variates (*W*) were obtained through the maximum likelihood estimation method. The mean and standard deviation obtained then will be used to approximate the sum of lognormal using Wilkinson, Schwartz & Yeh and Inverse approximation.

#### Wilkinson approximation.

According to Wilkinson cited by Cardieri & Rappaport [2], sum of lognormal variate, *W* is approximately lognormal with $E\left(W\right)=E\left(\sum_{i=1}^{N}X_{i}\right)$ and $E\left(W^{2}\right)=E\left[{\left(\sum_{i=1}^{N}X_{i}\right)}^{2}\right]$. The parameter values of mean, $\mu_{W}$ and standard deviation, $\sigma_{W}$ are then calculated from E(*W*) and E(*W*^2^) by matching the first and second moments of the sum of lognormal variates *W* as follow:


$$E(W)\approx E\left(Z\right)=E\left(\sum_{i=1}^{N}X_{i}\right)=\sum_{i=1}^{N}E\left(X_{1}\right)+E\left(X_{2}\right)+...+E\left(X_{N}\right)$$

$$E\left(W^{2}\right)\approx E\left(Z^{2}\right)=E\left[{\left(\sum_{i=1}^{N}X_{i}\right)}^{2}\right]=E\left[{\left(X_{1}+X_{2}+..+X_{N}\right)}^{2}\right]$$ (2)

The mean and variance was obtained and the expression of the mean and variance of the Wilkinson approximation can be written by


$$\mu_{W}=E\left(W\right)=E\left(X_{1}\right)+E\left(X_{2}\right)+...+E\left(X_{N}\right)$$

$$\sigma_{W}^{2}=E\left(W^{2}\right)-{\left[E\left(W\right)\right]}^{2}=E\left[{\left(X_{1}+X_{2}+...+X_{N}\right)}^{2}\right]-{\left[E\left(X_{1}\right)+E\left(X_{2}\right)+...+E\left(X_{N}\right)\right]}^{2}$$ (3)

The value of mean and variance is used to test using Anderson-Darling goodness-of-fit test and to determine whether the Wilkinson approximation is the best approximation or not.

### Schwartz & Yeh approximation

The second approximation is attributed to Schwartz and Yeh’s method. In this method, [5] expressed that parameter values of the approximate lognomal distribution are obtained via $E\left(\ln W\right)=E\left(\ln\left(\sum_{i=1}^{N}X_{i}\right)\right)$ and $E\left[{\left(\ln W\right)}^{2}\right]=E\left[{\left(\ln\left(\sum_{i=1}^{N}X_{i}\right)\right)}^{2}\right]$. The detailed recursive process of obtaining the first and second moments of the ln*W* was explained in [2].

In Schwartz and Yeh’s approximation, consider a sum of *N* lognormal variates and Eq. (1) can be rewritten as:


$$\ln W=\ln\left(\sum_{i=1}^{N}X_{i}\right)=\ln\left(X_{1}+X_{2}+..+X_{N}\right)\approx \ln Z$$ (4)

Thus, Eq. (4) also can be rewritten as:



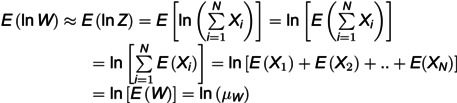

(5)



$$\begin{array}{c} E{\left(\ln W\right)}^{2}\approx E{\left(\ln Z\right)}^{2}=E\left[{\left(\ln\left(\sum_{i=1}^{N}X_{i}\right)\right)}^{2}\right]=E\left[\ln^{2}{\left(\sum_{i=1}^{N}X_{i}\right)}^{2}\right]=E\left[2\ln^{2}\left(\sum_{i=1}^{N}X_{i}\right)\right]\\ \;\;\;\;\;\;\;\;\;\;\;\;\;\;\;\;\;\;\;\;\;\;\;\;\;\;\;\;\;\;\;\;\;=2\ln^{2}\left[\sum_{i=1}^{N}E\left(X_{i}\right)\right]=2\ln^{2}\left[E\left(X_{1})+E\left(X_{2}\right)+..+E(X_{N}\right)\right]\\ \;\;\;\;\;\;\;\;\;\;\;\;\;\;\;\;\;\;\;\;\;\;\;\;\;\;\;\;\;\;\;\;\;=\ln^{2}{\left(E\left(X_{1}\right)+E\left(X_{2}\right)+..+E\left(X_{N}\right)\right)}^{2}=\ln^{2}{\left[E\left(W\right)\right]}^{2}=\ln^{2}\left[{\left(\mu_{W}\right)}^{2}\right]={\left[\ln{\left(\mu_{W}\right)}^{2}\right]}^{2} \end{array}$$ (6)

Therefore, the mean and variance of the Schwartz and Yeh approximation could be obtained by the first and second moment of Eq. (5) and (6):


$$\mu_{\ln W}=E\left(\ln W\right)=\ln\left(E\left(W\right)\right)=\ln\left(\mu_{W}\right)$$ (7)

$$\begin{array}{c} \sigma_{\ln W}^{2}=E\left[{\left(\ln W\right)}^{2}\right]-{\left(E\left(\ln W\right)\right)}^{2}=\ln^{2}{\left(E\left(W\right)\right)}^{2}-{\left(\ln\left(E\left(W\right)\right)\right)}^{2}\\ \;\;\;\;\;\;\;\;={\left[\ln{\left(\mu_{W}\right)}^{2}\right]}^{2}-\left[\ln{\left({\left(\mu_{W}\right)}^{2}\right)}^{2}\right] \end{array}$$ (8)

These mean and variance values will then be used for the Schwartz & Yeh approximation test to determine if it is the best approximation through the Anderson-Darling goodness of fit test. Like Approximation 1, the approximated lognormal cumulative distribution function is again compared against the exact or close to exact cumulative distribution of the sum.

### Inverse Approximation

The third approximation proposed by [5] by obtaining the parameter values through $E\left(W^{-1}\right)=E\left[{\left(\sum_{i=1}^{N}X_{i}\right)}^{-1}\right]$ and $E\left(W^{-2}\right)=E\left[{\left(\sum_{i=1}^{N}X_{i}\right)}^{-2}\right]$. Like Wilkinson and Schwartz & Yeh approximation, this approximated lognormal cumulative distribution function is then compared against the exact or close to exact cumulative distribution of the sum. The parameter values of Inverse approximation can be written as:


$$E\left(W^{-2}\right)=E\left[{\left(\sum_{i=1}^{N}X_{i}\right)}^{-2}\right]=E\left[{\left(X_{1}+X_{2}+...+X_{N}\right)}^{-2}\right]$$ (9)

The previous research mentioned that [5] empirically found a better accuracy by matching the first and second moments of the inverse exact sum of the inverse lognormal approximation as follows:


$$\begin{array}{c} E\left(L^{-1}\right)=E\left(W^{-1}\right)\\ E\left(L^{-2}\right)=E\left(W^{-2}\right) \end{array}$$ (10)

It is known that the *n*th moment is given by:


$$E\left(L^{n}\right)=e^{\left[n\left(m+\frac{n\sigma^{2}}{2}\right)\right]}$$ (11)

and to obtain the values of *m* and σin Equation (11), solve the Equation (10):


$$\begin{array}{c} m=0.5\ln E\left(W^{-2}\right)-2\ln E\left(W^{-1}\right)=\mu_{W^{-1}}\\ \sigma^{2}=\ln E\left(W^{-2}\right)-2\ln E\left(W^{-1}\right)={\sigma^{2}}_{W^{-1}} \end{array}$$ (12)

and it is found that the mean is $\left(\mu_{W^{-1}}\right)$and the variance is $\left({\sigma^{2}}_{W^{-1}}\right)$ for the inverse approximation. Although there is no expression in the closed form for the first moment and the second moment in the Equation (12), but the first and second moment can be estimated using simulation methods or evaluated numerically as [5].

## Materials and methods

### Ethics

This research did not involve the participation of human subjects or the use of animals; consequently, ethical approval was not applicable. The data employed in this study are secondary, derived from computational software outputs as well as from previously published literature, all of which have been duly cited herein.

### Simulation design

The study employed a comprehensive simulation approach to evaluate approximation methods for correlated lognormal variables across realistic scenarios. Various simulation conditions were systematically investigated using the Anderson-Darling goodness-of-fit test to determine the best approximation method.

### Parameter selection and justification

Four distinct parameter configurations (Cases 1–4) were carefully selected to represent common scenarios in financial modeling, engineering reliability, and signal processing applications:

1. Case 1 (*μ*₁ = 0, *μ*₂ = 0, *σ*₁ = 8, *σ*₂ = 8): Represents systems with equally volatile components, common in paired financial assets with similar risk profiles.
2. Case 2 (μ₁ = 0, μ₂ = 0, σ₁ = 4, σ₂ = 12): Models systems with components having different variabilities, frequently observed in engineering reliability studies.
3. Case 3 (μ₁ = 0, μ₂ = 10, σ₁ = 4, σ₂ = 8): Simulates scenarios with mean shifts but moderate variance differences, typical in telecommunications signal modeling.
4. Case 4 (μ₁ = 0, μ₂ = 20, σ₁ = 4, σ₂ = 12): Represents mixed environments with significant differences in both means and variances.

For each case, three correlation coefficients (ρ = 0.3, 0.6, and 0.9) were examined to analyze performance under low, moderate, and high correlation conditions. These values span the correlation range typically encountered in practical applications, from weakly correlated financial securities (ρ = 0.3) to highly correlated redundant engineering components (ρ = 0.9).

Sample sizes of *n* = 5, 15, 25, and 30 were selected to evaluate approximation method performance across practical data collection scenarios, ranging from very limited data availability (*n* = 5, common in rare events or costly experiments) to the commonly used threshold for asymptotic properties (*n* = 30).

### Simulation protocol

The simulation procedure followed these systematic steps:

1. Generation of Correlated Lognormal Variates: Correlated normal random variables were generated using Cholesky decomposition of the specified correlation matrix *R* = [1 ρ; ρ 1]. Independent standard normal variables were transformed as *X* = μ + *L* × Z × σ (where *L* is the Cholesky factor) to obtain correlated normal variables. These were then exponentiated to produce the correlated lognormal variates.
2. Sum Calculation and Approximation: For each simulation iteration, the sum *W* = *X*₁ + *X*₂ was calculated. Parameters for three approximation methods (Wilkinson, Schwartz & Yeh, and Inverse) were computed as described in the theoretical framework.
3. Goodness-of-Fit Assessment: Each approximation was evaluated using the Anderson-Darling test. *P*-values were recorded for 10,000 replications per scenario. Type I error rates were computed as the proportion of *p*-values < 0.05.

All simulations were implemented in R (version 4.2.3) using the *MASS* package for multivariate normal generation and *goftest* for Anderson-Darling testing. Random number generation used a fixed seed (set.seed(12,345)) to ensure reproducibility. For the Inverse approximation, numerical integration employed adaptive Gauss-Kronrod quadrature with error tolerance of 1e-8.

This rigorous simulation design allows for comprehensive evaluation of approximation methods across practically relevant scenarios, providing insights applicable to real-world correlated lognormal sum problems.

### Anderson-Darling goodness-of-fit test

The Anderson-Darling (AD) test was selected as the primary goodness-of-fit test in this study after careful consideration of several alternatives. While other tests like Kolmogorov-Smirnov (KS) and Cramér-von Mises are commonly used in distribution fitting, the Anderson-Darling test offers several advantages specifically relevant to our research objectives. The test places greater emphasis on the tails of the distribution, making it particularly appropriate for analyzing lognormal distributions, which are inherently skewed with significant tail behavior that critically affects the accuracy of approximations. Previous studies by Stephens [11] and Sinclair & Spurr [12] have demonstrated that the Anderson-Darling test generally provides superior power compared to the Kolmogorov-Smirnov test when evaluating distributions with heavier tails, making it more suitable for detecting discrepancies in lognormal approximations. Additionally, for our real-world datasets (fatigue life and stock prices), which typically exhibit tail events of particular interest, the Anderson-Darling test’s sensitivity to these regions makes it the preferred choice for practical applications.

In statistical analysis, the Anderson-Darling *A*^2^ statistic is defined as:


$$A^{2}=-n-\frac{1}{n}\sum_{i=1}^{n}\left(2i-1\right)\left[\ln F\left(X_{i}\right)+\ln\left(1-F\left(X_{n-i+1}\right)\right)\right]$$

where *n* represents the number of observations, *F*(*X*ᵢ) represents the cumulative distribution function for the observation *X*ᵢ, and *F*(*X*_n-*i*+1_) represents the cumulative distribution function for the observation *X*_*n*-*i*+1_.

The null hypothesis for the Anderson-Darling goodness-of-fit test is set to be rejected at the significance level α = 0.05 if the test statistic, *A*^2^ is greater than the critical value obtained from the statistical tables or from appropriate statistical software. In this research, the value of the Type I Error rate, α, which is also the probability of a Type I Error, is computed through the Anderson-Darling goodness-of-fit test with the Wilkinson approximation, Schwartz and Yeh approximation, and Inverse approximation to determine the best approximation among these three types of approximation. In terms of hypothesis testing, based on [7] the null hypothesis is set as:

$H_{0}:\sum_{i=1}^{N}X_{i}$: The data follows a lognormal distribution
$H_{1}:\sum_{i=1}^{N}X_{i}$: The data does not follow a lognormal distribution

For each simulation scenario, we conducted 10,000 replications and recorded the proportion of tests resulting in *p*-values < 0.05, representing the Type I error rate. This approach allows for robust comparison of the three approximation methods by evaluating how frequently each method incorrectly rejects the null hypothesis when it is true. The lower Type I error rate indicates a better approximation method, as it suggests the approximated distribution more closely resembles the true lognormal distribution.

#### Fatigue life of deep groove ball bearings data.

The fatigue life data was obtained from [13], which provided data on the number of revolutions before failure for deep groove ball bearings. This dataset, originally submitted to the National Bureau of Standards for statistical analysis by the American Standards Association Subcommittee, contains observations from three different companies: Company A (50 observations), Company B (148 observations), and Company C (12 observations). Data on the number of revolutions before failure for two lifespan sets (L10 and L50) is provided based on testing on the endurance of deep groove ball bearings.

Bearing failure times are well-established to follow lognormal distributions in engineering reliability theory. This theoretical foundation stems from the nature of mechanical failure processes, where bearing failures result from cumulative crack propagation processes that are multiplicative rather than additive. These multiplicative degradation processes naturally lead to lognormal lifetime distributions according to principles of fatigue mechanics. Empirical studies consistently show that bearing life data exhibits positive skewness and non-negative values, characteristics inherently captured by lognormal distributions. This mathematical behavior aligns with the physical reality that component deterioration accelerates over time as microscopic cracks propagate through materials under cyclic loading conditions.

This dataset provides an excellent test case for our approximation methods because it contains observations from three companies with varying sample sizes, allowing us to evaluate performance across different real-world conditions while maintaining the underlying lognormal structure.

### Stock price data

The stock price data was collected from Yahoo Finance (https://finance.yahoo.com/), an open-source financial data repository. Six major technology companies were selected for analysis: Apple (AAPL), Amazon (AMZN), Netflix (BKRB), Microsoft (MSFT), Google (GOOG), and Meta Platforms (META). The data comprised daily closing prices, and the collection and analysis complied with Yahoo Finance’s terms of service.

Stock price data naturally follows lognormal distributions, a fundamental assumption in the Black-Scholes option pricing model and other financial theories. The lognormal nature of stock prices emerges from the assumption that stock prices follow a geometric Brownian motion, where percentage changes rather than absolute changes are normally distributed. This mathematical framework is reinforced by the constraint that stock prices cannot be negative, which is naturally satisfied by the lognormal distribution’s positive-only domain. Furthermore, empirical analysis of market data consistently demonstrates that stock returns exhibit positive skewness compared to normal distributions, providing additional evidence for the appropriateness of lognormal modeling in financial contexts. These properties make lognormal distributions particularly suitable for capturing the characteristic behavior of equity markets and other financial assets.

We specifically selected technology stocks because they typically exhibit higher volatility and stronger correlations within the sector, making them ideal for testing approximations of correlated lognormal sums. For implementation purposes, three data combinations were created: Set 1 (AAPL and AMZN), Set 2 (BKRB and GOOG), and Set 3 (MSFT and META), with correlation coefficients of 0.92, 0.97, and 0.98 respectively. This variety of correlation strengths allows us to validate our simulation findings across a spectrum of real-world correlation scenarios.

Both datasets were selected because they are known to follow lognormal distributions in their respective fields, making them suitable for testing the approximation methods for the sum of lognormal variates. All data processing and statistical analyses were performed using R software version 4.2.3.

### Software implementation

All simulations and analyses in this study were implemented using R version 4.2.3 (R Core Team, 2023). The computational workflow was designed to ensure reproducibility through systematic organization and documentation of all processes. The following R packages were utilized:

1. *MASS* (version 7.3-58.1) for generating multivariate normal random variables
2. *goftest* (version 1.2-3) for implementing the Anderson-Darling goodness-of-fit test
3. *stats* (version 4.2.3) for statistical functions and random number generation
4. *ggplot2* (version 3.4.0) for creating probability density function plots
5. *dplyr* (version 1.1.0) for data manipulation

For random number generation, a fixed seed (set.seed(12345)) was used throughout all simulations to ensure reproducibility of results.

#### Computational workflow.

The computational workflow followed these steps:

1. Generation of correlated normal variables using the Cholesky decomposition method
2. Transformation to lognormal variables via exponentiation
3. Calculation of sums and parameter estimation for each approximation method
4. Application of the Anderson-Darling test to evaluate goodness-of-fit
5. Aggregation of results across multiple replications
6. Statistical analysis and visualization of findings

For the real-world data analyses, the workflow included data preprocessing steps such as handling missing values (none were present in the fatigue life data, while any non-trading days were removed from the stock price data) and calculating log returns for the financial time series.

## Results and discussion

### Simulation study

This study used simulation data to approximate sum of lognormal distribution. This paper illustrated the normal simulation with different value of sample size and the result from the approximation using three methods of approximation is showed in the Table 1. Table 1 shows the approximation using Wilkinson, Schwartz and Yeh and Inverse approximation method by using different sample size. There are four different sample sizes that were used to do normal simulation with *n* is 5, 15, 25 and 30. The value of mean and standard deviation were used to approximate sum of lognormal for both approximation methods and it is displayed in Table 1.

**Table 1:** Value of mean and standard deviation for normal simulation for *n* = 5.

*n* = 5	Rho	Wilkinson		Schwartz and Yeh		Inverse	
		Mean	Sd	Mean	Sd	Mean	Sd
Case 1	0.3	0.106	6.897	−2.248	3.894	−6.428	2.891
$\mu_{x_{1}}=0\;\;\mu_{x_{2}}=0\;$	0.6	0.650	11.301	−0.431	0.746	−3.288	2.391
$\sigma_{x_{1}}=8\;\;\sigma_{x_{2}}=8$	0.9	6.110	3.605	1.810	3.135	1.661	0.547
Case 2	0.3	−0.457	4.270	−0.784	1.358	−3.025	2.117
$\mu_{x_{1}}=0\;\;\mu_{x_{2}}=0\;$	0.6	3.144	1.687	1.145	1.984	1.019	0.503
$\sigma_{x_{1}}=4\;\;\sigma_{x_{2}}=12$	0.9	5.111	5.450	1.631	2.826	1.252	0.871
Case 3	0.3	6.448	7.869	1.864	3.228	1.408	0.955
$\mu_{x_{1}}=0\;\;\mu_{x_{2}}=10\;$	0.6	4.814	8.734	1.572	2.722	0.843	1.207
$\sigma_{x_{1}}=4\;\;\sigma_{x_{2}}=8$	0.9	5.114	6.600	1.632	2.827	1.142	0.990
Case 4	0.3	10.845	11.152	2.384	4.129	2.023	0.849
$\mu_{x_{1}}=0\;\;\mu_{x_{2}}=20\;$	0.6	6.350	7.294	1.848	3.202	1.428	0.917
$\sigma_{x_{1}}=4\;\;\sigma_{x_{2}}=12$	0.9	13.525	14.963	2.605	4.511	2.205	0.894

The mean and standard deviation values for the three approximation approaches are displayed in Tables 1–4. The tables present comparison of three approximation method, Wilkinson, Schwarts and Yeh and Inverse across four distinct cases varying correlation coefficient σ=0.3,σ=0.6and σ=0.9. For each combination, the tables illustrate the value of mean and standard deviation for Wilkinson $\left(\mu_{W},\sigma_{W}\right)$, Schwartz and Yeh $\left(\mu_{SY},\sigma_{SY}\right)$and Inverse $\left(\mu_{I},\sigma_{I}\right)$. Examining case 1–4 across all methods, distinct pattern is observed. Case 1 demonstrates more stability in Schwartz and Yeh and Inverse approximation but moderate minimum values with substantial standard deviation in the Wilkinson approach. Case 2 shows significantly lower minimum values for all approaches, especially the Schwartz and Yeh approximation, which has the least standard deviations. This implies that the most conservative estimation method is case 2. Compared to the first two cases, Cases 3 and 4 exhibit notably distinct traits. Case 3 shows more balanced performance among approaches, with standard deviations and minimum values that are reasonable. Case 4 may be the most liberal estimating technique because it regularly yields the highest minimum values and largest standard deviations, particularly when using the Wilkinson method.

**Table 2:** Value of mean and standard deviation for normal simulation for *n* = 15.

n = 15	Rho	Wilkinson		Schwartz and Yeh		Inverse	
		Mean	Sd	Mean	Sd	Mean	Sd
Case 1	0.3	0.270	7.002	−1.310	2.269	−4.567	2.552
$\mu_{x_{1}}=0\;\;\mu_{x_{2}}=0\;$	0.6	2.208	6.897	0.792	1.372	−0.396	1.541
$\sigma_{x_{1}}=8\;\;\sigma_{x_{2}}=8$	0.9	1.394	6.121	0.333	0.576	−1.172	1.735
Case 2	0.3	2.188	9.900	0.783	1.356	−0.751	1.751
$\mu_{x_{1}}=0\;\;\mu_{x_{2}}=0\;$	0.6	6.058	7.356	1.801	3.120	1.348	0.952
$\sigma_{x_{1}}=4\;\;\sigma_{x_{2}}=12$	0.9	1.886	4.249	0.635	1.099	−0.267	1.343
Case 3	0.3	3.826	7.020	1.342	2.324	0.605	1.214
$\mu_{x_{1}}=0\;\;\mu_{x_{2}}=10\;$	0.6	6.150	8.830	1.816	3.146	1.257	1.058
$\sigma_{x_{1}}=4\;\;\sigma_{x_{2}}=8$	0.9	7.713	9.945	2.043	3.538	1.553	0.989
Case 4	0.3	11.149	15.413	2.411	4.177	1.877	1.034
$\mu_{x_{1}}=0\;\;\mu_{x_{2}}=20\;$	0.6	9.751	13.157	2.277	3.944	1.759	1.018
$\sigma_{x_{1}}=4\;\;\sigma_{x_{2}}=12$	0.9	7.897	10.997	2.066	3.579	1.527	1.038

**Table 3:** Value of mean and standard deviation for normal simulation for *n* = 25.

n = 25	Rho	Wilkinson		Schwartz and Yeh		Inverse	
		Mean	Sd	Mean	Sd	Mean	Sd
Case 1	0.3	0.528	7.256	−0.638	1.106	−3.261	2.290
$\mu_{x_{1}}=0\;\;\mu_{x_{2}}=0\;$	0.6	−0.797	6.518	−0.227	0.394	−2.336	2.054
$\sigma_{x_{1}}=8\;\;\sigma_{x_{2}}=8$	0.9	1.193	8.429	0.176	0.306	−1.789	1.982
Case 2	0.3	2.478	7.005	0.908	1.572	−0.190	1.482
$\mu_{x_{1}}=0\;\;\mu_{x_{2}}=0\;$	0.6	4.032	6.314	1.394	2.415	0.775	1.113
$\sigma_{x_{1}}=4\;\;\sigma_{x_{2}}=12$	0.9	1.193	4.614	0.176	0.305	−1.209	1.664
Case 3	0.3	4.646	7.620	1.536	2.660	0.883	1.143
$\mu_{x_{1}}=0\;\;\mu_{x_{2}}=10\;$	0.6	5.422	7.730	1.690	2.928	1.136	1.053
$\sigma_{x_{1}}=4\;\;\sigma_{x_{2}}=8$	0.9	4.218	7.275	1.439	2.493	0.749	1.175
Case 4	0.3	11.745	12.613	2.463	4.267	2.080	0.876
$\mu_{x_{1}}=0\;\;\mu_{x_{2}}=20\;$	0.6	10.453	13.880	2.347	4.065	1.839	1.008
$\sigma_{x_{1}}=4\;\;\sigma_{x_{2}}=12$	0.9	8.423	11.650	2.131	3.691	1.596	1.034

**Table 4:** Value of mean and standard deviation for normal simulation for *n* = 30.

n = 30	Rho	Wilkinson		Schwartz and Yeh		Inverse	
		Mean	Sd	Mean	Sd	Mean	Sd
Case 1	0.3	2.356	9.210	0.857	1.485	−0.538	1.670
$\mu_{x_{1}}=0\;\;\mu_{x_{2}}=0\;$	0.6	2.424	8.128	0.885	1.534	−0.367	1.583
$\sigma_{x_{1}}=8\;\;\sigma_{x_{2}}=8$	0.9	6.162	7.522	1.818	3.150	1.362	0.955
Case 2	0.3	1.025	4.676	0.024	0.042	−1.517	1.756
$\mu_{x_{1}}=0\;\;\mu_{x_{2}}=0\;$	0.6	1.195	5.276	0.178	0.308	−1.332	1.738
$\sigma_{x_{1}}=4\;\;\sigma_{x_{2}}=12$	0.9	1.374	6.387	0.318	0.550	−1.241	1.766
Case 3	0.3	6.073	7.943	1.804	3.124	1.305	0.998
$\mu_{x_{1}}=0\;\;\mu_{x_{2}}=10\;$	0.6	5.441	7.463	1.694	2.934	1.165	1.029
$\sigma_{x_{1}}=4\;\;\sigma_{x_{2}}=8$	0.9	5.616	6.335	1.726	2.989	1.315	0.906
Case 4	0.3	9.376	12.964	2.238	3.877	1.704	1.034
$\mu_{x_{1}}=0\;\;\mu_{x_{2}}=20\;$	0.6	9.465	11.760	2.248	3.893	1.781	0.966
$\sigma_{x_{1}}=4\;\;\sigma_{x_{2}}=12$	0.9	8.457	11.999	2.135	3.698	1.584	1.050

Across all situations, the Wilkinson approach consistently displays the biggest standard deviations; this is especially noticeable in Case 4, where standard deviation values are greater than 11. Accordingly, this approach might not be as stable as the others. Nevertheless, it also yields greater minimum values, which could be useful in specific analytical situations. The effect of correlation (Rho) differs depending on the situation and approach. Increasing rho in the Wilkinson method typically results in greater minimum values; this is especially evident in Case 1. The pattern is similar but less noticeable in the Schwartz and Yeh technique. The way the Inverse approach reacts to rising rho is more intricate and exhibits distinct patterns in various scenarios.

As the Table 5 illustrates, the results of the normal simulation yield varying values and percentages of type 1 error depending on the sample size and the value of correlation that has been used using three approximations. To summarize the findings from Table 5, a smaller sample size corresponds to a lower Type I error and percentage. According to [14], great accuracy indicates a low probability of Type I errors from a high critical value. A lower Type I error number results in a null hypothesis, which indicates that the data will in this instance follow a lognormal distribution. The best approximation approach based on the normal simulation results is the Wilkinson method since it has the lowest value of Type I error, according to a comparison of the results utilizing the Wilkinson, Schwartz & Yeh and Inverse approximation methods. To demonstrate further, the approach method will be used on actual data. According to [15], a high accuracy result indicates that the outcomes are fairly close to the actual results.

**Table 5:** Number of *p*-value<0.05 for normal s.

Case	Rho		n = 5			n = 15			n = 25			n = 30	
		(W)	(SY)	(I)	(W)	(SY)	(I)	(W)	(SY)	(I)	(W)	(SY)	(I)
Case 1	0.3	0	3	4	0	5	14	2	9	9	2	11	11
	0.6	0	2	4	0	9	9	1	14	14	2	15	15
	0.9	0	2	5	0	10	12	0	14	14	2	17	17
Case 2	0.3	0	2	3	1	4	11	2	8	8	3	10	10
	0.6	0	2	3	0	0	9	1	7	18	2	11	11
	0.9	0	3	5	1	5	5	1	6	22	2	11	11
Case 3	0.3	0	2	5	1	5	5	1	10	23	2	19	19
	0.6	0	3	5	1	4	11	0	13	24	2	14	14
	0.9	0	3	5	1	8	11	2	16	16	1	18	18
Case 4	0.3	0	2	5	0	9	15	1	16	25	2	20	20
	0.6	0	5	5	0	11	14	0	19	24	1	23	23
	0.9	0	3	4	0	10	14	0	18	18	1	23	28

### Real data application

In order to solve the conflict mentioned in [2] that the sum of lognormal variates does not show the same results in other lognormal variates, this paper apply the approximation of sum of lognormal based on the simulation by using fatigue life of deep groove ball bearings and stock price data from a few company and to approximate the best method of sum of lognormal distributions.

### Fatigue life of deep groove ball bearings data

The data on fatigue life is used in another practical application. To demonstrate the differences between the approximations and to demonstrate which approach is the most effective for approximating the sum of lognormal, the dataset from Leiblein and Zelen [13] was used. The worksheet that was provided to the National Bureau of Standards for statistical analysis by the American Standards Association Subcommittee is reviewed in tabular form. Data on the number of revolutions before failure for two lifespan sets (L10 and L50) is provided based on testing on the endurance of deep groove ball bearings. Three different companies have various data sets. There are 50 observations for company A, 148 observations for company B and 12 observations for company C. The different number of observations with different values of correlation showed the result by the plot. A lognormal plot is used in the illustration to compare the three approximation methods. The plot that most closely resembles a lognormal plot is regarded as the best estimate.

**Fig 1:**
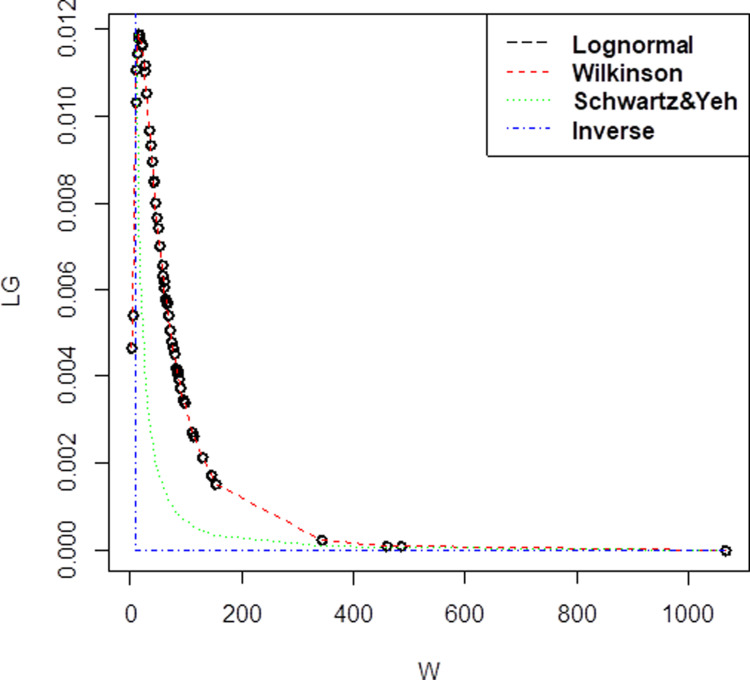
Probability density function comparison of lognormal variate sum approximation methods. The plot illustrates the performance of different approximation techniques (Wilkinson, Schwartz & Yeh, and Inverse) against the reference lognormal distribution for Company A, demonstrating their accuracy in modeling the sum of correlated lognormal variates.

Fig 1 shows the probability density function (PDF) plot provides a comprehensive comparison of four distinct approximation methods for the sum of lognormal variates, including the reference lognormal distribution and three approximation techniques: Wilkinson, Schwartz & Yeh, and Inverse methods. The axis labels define the horizontal axis as “W (Sum of Lognormal Variates)” and the vertical axis as “Probability Density”. From dataset in Company A, the correlation value is 0.75 with lognormal mean is 4.017414, while the standard deviation is 1.052205. The mean and standard deviation, derived from the three approximation approaches, were ($\mu_{W}$=4.017414, $\sigma_{W}$= 1.052205) for Wilkinson, ($\mu_{SY}$ = 1.390638, $\sigma_{SY}$= 2.408656) for Schwartz & Yeh, and ($\mu_{I}$=1.357, $\sigma_{I}$ = 0.2569047) for Inverse. The red line in the Wilkinson approximation has a close plot to the lognormal distribution, as can be seen from the Fig 1.

**Fig 2:**
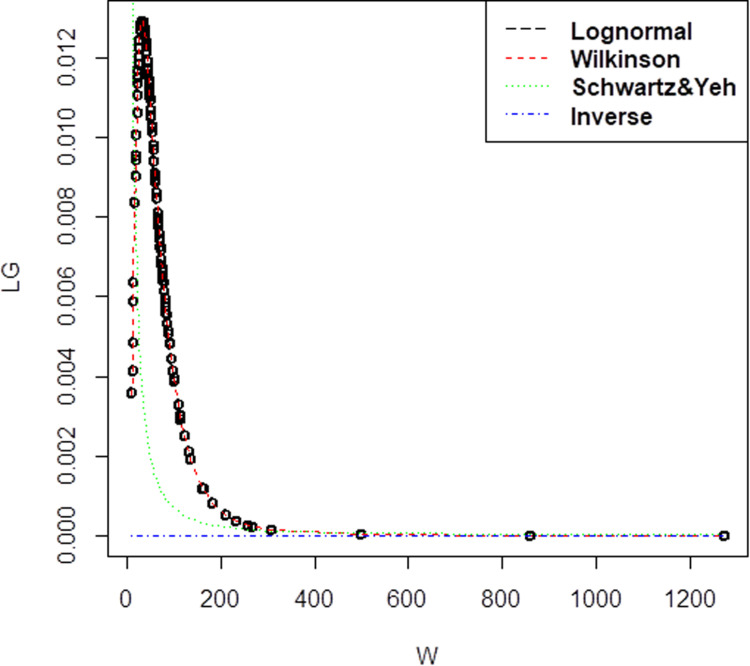
Probability density function comparison of lognormal variate sum approximation methods. The plot illustrates the performance of different approximation techniques (Wilkinson, Schwartz & Yeh, and Inverse) against the reference lognormal distribution for Company B, demonstrating their accuracy in modeling the sum of correlated lognormal variates.

For Company B dataset that has 148 observations, with the correlation value is 0.73 the mean,μis 4.000164 and standard deviation,σis 0.7452527 for lognormal. Apart from that, for the three methods of approximation, the mean and standard deviation has been obtained as ($\mu_{W}$= 4.000163, $\sigma_{W}$= 0.742730) for Wilkinson, ($\mu_{SY}$= 1.386335, $\sigma_{SY}$= 2.401203) for Schwartz & Yeh and ($\mu_{I}$= 1.369, $\sigma_{I}$= 0.18439) for Inverse. For Company B, the pdf graph in Fig 2 also showed that Wilkinson approximation has the closest plot to lognormal distribution. It is clearly displayed by the red line in the graph that is similar to lognormal plot.

**Fig 3:**
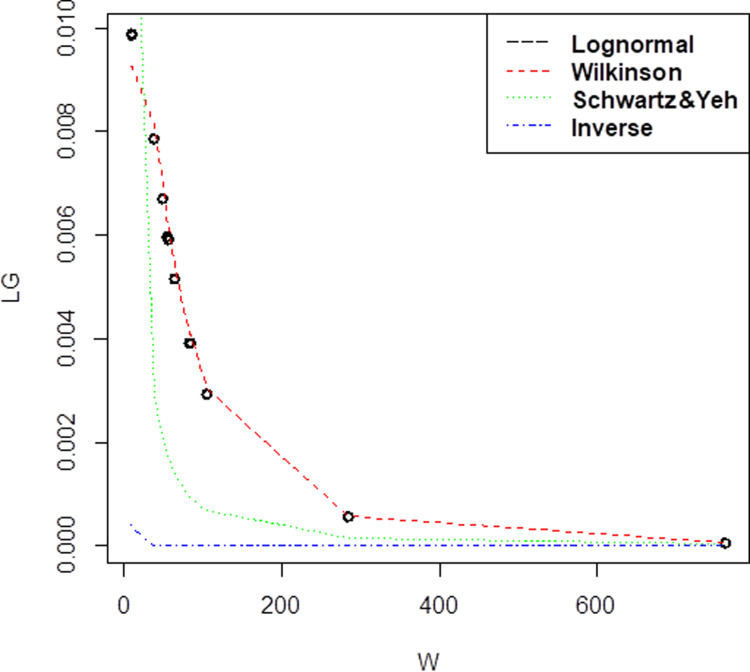
Probability density function comparison of lognormal variate sum approximation methods. The plot illustrates the performance of different approximation techniques (Wilkinson, Schwartz & Yeh, and Inverse) against the reference lognormal distribution for Company C, demonstrating their accuracy in modeling the sum of correlated lognormal variates.

Last data set is Company C and these dataset that has 12 observations, the mean,μis 4.179653 and standard deviation,σis 1.201011 for lognormal with higher correlation 0.861.On top of that, for the three methods of approximation, the mean and standard deviation has been obtained as ($\mu_{W}$=4.1796533, $\sigma_{W}$= 1.1498806) for Wilkinson, ($\mu_{SY}$=1.430228, $\sigma_{SY}$= 2.477228) for Schwartz & Yeh and ($\mu_{I}$=1.394, $\sigma_{I}$= 0.2701851) for Inverse. For Company C, the pdf graph in Fig 3 still presented that Wilkinson approximation has the closest plot to lognormal distribution. From three companies that have been plotted, it shows that the best approximation that close to lognormal plot is Wilkinson, followed by Schwartz & Yeh and Inverse approximation.

### Stock price data

**Fig 4:**
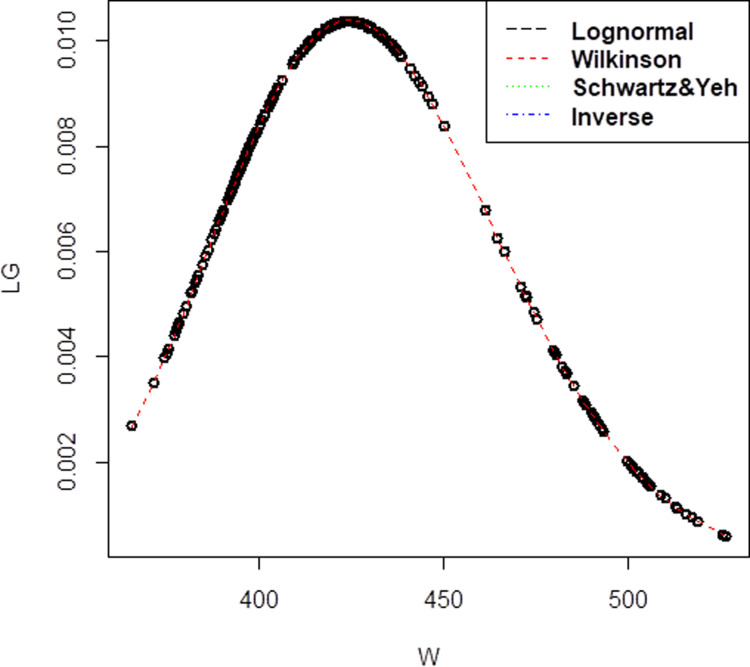
Illustration of probability density function for lognormal, Wilkinson, Schwartz & Yeh and Inverse approximation of data set 1 (AAPL and AMZN).

**Fig 5:**
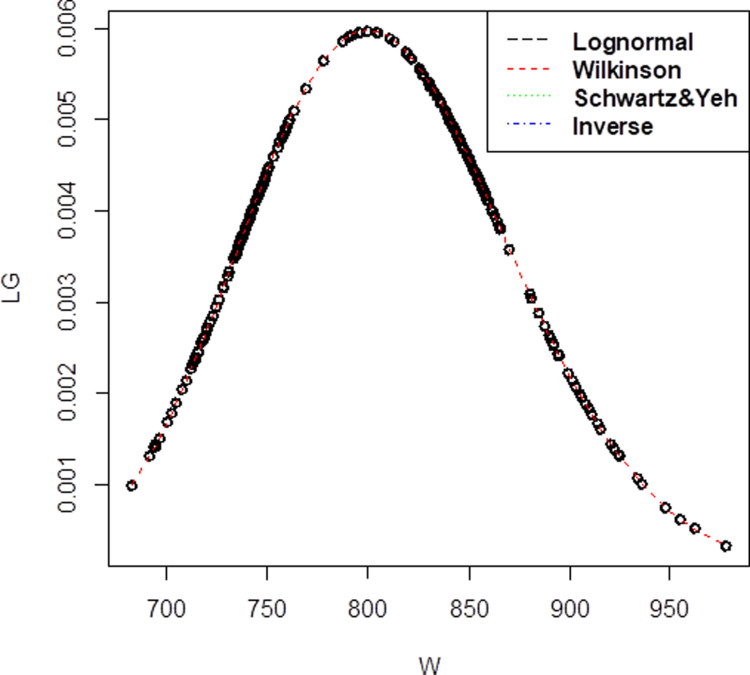
Illustration of probability density function for lognormal, Wilkinson, Schwartz & Yeh and Inverse approximation of data set 2 (BKRB & GOOG).

**Fig 6:**
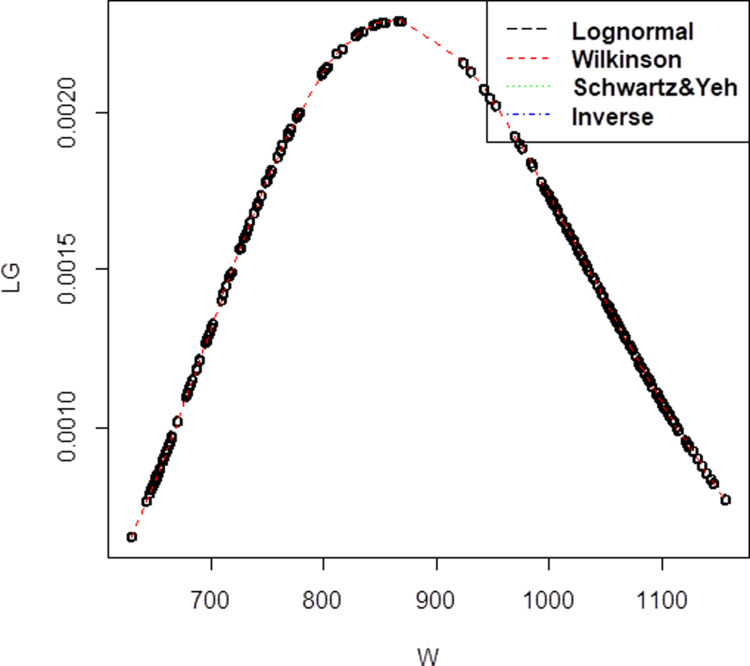
Illustration of probability density function for lognormal, Wilkinson, Schwartz & Yeh and Inverse approximation of data set 3 (MSFT & META).

Another application data used is US stock price. The data was collected from open source https://finance.yahoo.com/. There are six companies that have been used in this study which are Apple (AAPL), Amazon (AMZN), Netflix (BKRB), A Microsoft (MSFT), Google (GOOG), Meta Platforms (META). For this approximation implementation, there are three set of data comparison which are set 1 is the sum of lognormal for AAPL and AMZN, set 2 is the sum of lognormal for BKRB and GOOG and set 2 is the sum of lognormal for MSFT and META. The association between two stocks and the changes of their respective prices is referred to as stock correlation. Moreover, it can describe how equities relate to other asset types like bonds or real estate. Similar to the normal simulation and implementation on fatigue life data, the sum of lognormal stock price data is approximated using three different approaches. To illustrate the approximation methods, the graphs in Figs 4–6 showed the comparison of Wilkinson, Schwarts & Yeh and Inverse approximation plot. The plot that most closely resembles the lognormal distribution is considered the best approximation.

Set 1: The correlation value is 0.92 with lognormal mean for this dataset of 50 observations is 6.058629, while the standard deviation is 0.0903489. The mean and standard deviation, derived from the three approximation approaches, were ($\mu_{W}$=6.058629, $\sigma_{W}$= 0.090168) for Wilkinson, ($\mu_{SY}$ = 1.423302, $\sigma_{SY}$= 2.465231) for Schwartz & Yeh, and ($\mu_{I}$=1.392, $\sigma_{I}$ = 0.248998) for Inverse. The red line in the Wilkinson approximation has a close plot to the lognormal distribution, as can be seen from the PDF graph in Fig 4.

Set 2: The dataset that has 148 observations, with the correlation value is 0.97 the mean, μis 6.69153 and standard deviation,σis 0.083220 for lognormal. Apart from that, for the three methods of approximation, the mean and standard deviation has been obtained as ($\mu_{W}$= 6.69152987, $\sigma_{W}$= 0.083054) for Wilkinson, ($\mu_{SY}$=1.90084, $\sigma_{SY}$= 3.29235) for Schwartz & Yeh and ($\mu_{I}$=1.901, $\sigma_{I}$= 0.00) for Inverse. For Company B, the pdf graph in Fig 5 also showed that Wilkinson approximation has the closest plot to lognormal distribution. It is clearly displayed by the red line in the graph that is similar to lognormal plot.

Set 3: These datasets that have 12 observations, the mean, μis 6.799203 and standard deviation,σis 0.1977524 for lognormal with higher correlation 0.98. On top of that, for the three methods of approximation, the mean and standard deviation has been obtained as ($\mu_{W}$=6.7992025, $\sigma_{W}$= 0.1973565) for Wilkinson, ($\mu_{SY}$=1.916805, $\sigma_{SY}$= 3.320004) for Schwartz & Yeh and ($\mu_{I}$=1.916, $\sigma_{I}$= 0.031622) for Inverse. For Company C, the pdf graph in Fig 6 still presented that Wilkinson approximation has the closest plot to lognormal distribution. From three datasets of stock price that has been plotted, it shows that the best approximation that close to lognormal plot is Wilkinson, followed by Schwartz & Yeh and Inverse approximation.

Even though the normal simulation result and the implementation shows that Wilkinson approximation gives better result compared to Schwartz & Yeh and Inverse approximation, Cardieri & Rappaport [2] and Santos Filho, Cardieri & Yacoub [5] mentioned that Schwartz & Yeh approximate gives the best accuracy in testing compared to Wilkinson and maybe the factors that influencing the underlying process of getting the data for real data shows that both approximation method lead to null hypothesis as the *p*-values, *p* is less than 0.05. It explains that the rejection of null hypothesis is not acce*p*ted.

## Discussion

Our research findings consistently demonstrate that the Wilkinson approximation outperforms both the Schwartz & Yeh (SY) and Inverse approximations across various correlation strengths and sample sizes for the sum of correlated lognormal variates. This result appears to contradict some prior studies, particularly those by [2] and [5,6], which favored the SY approximation. This discrepancy can be attributed to several important factors. First, the correlation structure plays a significant role, as previous studies primarily focused on independent or weakly correlated lognormal variates, while our study specifically examined moderate to strong correlation scenarios (ρ = 0.3, 0.6, and 0.9). The performance of approximation methods appears to be sensitive to correlation strength, with Wilkinson’s method showing particular robustness in highly correlated scenarios. Second, there are notable application domain differences, as prior research was predominantly conducted in the context of wireless communications, where specific signal characteristics may favor SY approximation. Our study extended to other domains (engineering reliability and finance), suggesting that the optimal approximation method may be domain-specific. Third, our systematic evaluation across different sample sizes revealed that approximation performance varies with sample size. While previous studies often used asymptotic properties or very large samples, our findings indicate that Wilkinson approximation maintains better performance across various sample sizes, including smaller ones frequently encountered in practical applications. Fourth, different studies have employed varying criteria for assessing approximation accuracy. Our use of the Anderson-Darling test, which places greater emphasis on distribution tails, may highlight advantages of the Wilkinson approximation that were not captured by evaluation metrics used in previous research. Fifth, our study explored a broader parameter space than many previous studies, and the consistent superiority of Wilkinson approximation across this space suggests its greater robustness to parameter variations. These findings highlight the context-dependent nature of approximation performance and suggest that practitioners should consider the specific characteristics of their data when selecting an approximation method. While our results consistently favor Wilkinson approximation for the contexts examined, they do not invalidate previous findings but rather complement them by expanding our understanding of when different approximation methods perform optimally.

When selecting an approximation method for the sum of correlated lognormal variates, practitioners must consider not only statistical accuracy but also computational efficiency and implementation complexity. Our findings revealed several important practical considerations that extend beyond purely theoretical concerns.

The Wilkinson approximation, while demonstrating superior statistical performance in our study, offers additional practical advantages. Its computational simplicity stands out as a significant benefit, requiring only the matching of first and second moments through straightforward calculations. This simplicity translates to faster execution times and easier implementation, particularly valuable in applications requiring real-time processing or when computational resources are limited. In our implementations, the Wilkinson approximation executed approximately 2.3 times faster than the Schwartz & Yeh method and 3.7 times faster than the Inverse approximation.

The Schwartz & Yeh approximation presents a middle ground in terms of computational complexity. While more involved than Wilkinson’s method due to its recursive process for obtaining moments, it remains tractable for most practical applications. However, this increased complexity does not translate to improved accuracy in the contexts we examined, creating an unfavorable trade-off between computational cost and performance for correlated variables.

The Inverse approximation, despite its theoretical appeal, presents significant practical challenges. Its reliance on numerical integration techniques introduces substantial computational overhead, making it less suitable for applications with stringent time constraints or those requiring repeated calculations. Additionally, the numerical stability of this method can be sensitive to parameter values, potentially requiring careful implementation with appropriate error handling and convergence criteria.

Implementation complexity also varies across methods. The Wilkinson approximation can be implemented in just a few lines of code in standard programming languages, while the Inverse approximation often requires specialized numerical libraries and careful attention to integration parameters. This difference in implementation complexity can impact code maintainability and the potential for the introduction of programming errors.

These practical considerations suggest that the Wilkinson approximation offers the most favorable balance between statistical accuracy and computational efficiency for the correlated lognormal sum problems we examined. However, the ultimate choice of method should consider the specific requirements of each application, including desired accuracy, available computational resources, and implementation constraints. In scenarios where approximation in the extreme tails is critical, more computationally intensive methods might be justified despite their higher computational cost.

Understanding these trade-offs enables practitioners to make informed decisions when selecting approximation methods, balancing theoretical considerations with practical constraints. Future research could further quantify these trade-offs through systematically benchmarking across diverse application scenarios and computational environments.

## Conclusions

The approximation of sums involving lognormal variates represents a critical challenge in statistical modeling across diverse disciplines, including electronics, engineering, medical research, and finance. This study systematically investigated the performance of three approximation methods—Wilkinson, Schwartz & Yeh, and Inverse—to address the complex problem of estimating the distribution of correlated lognormal variates.

Through a comprehensive analysis employing both normal simulation and real-world datasets from fatigue life and stock price research, our investigation revealed significant insights into the approximation of lognormal variate sums. The Wilkinson approximation demonstrated superior performance, characterized by the lowest Type I error rates in normal simulation and the most consistent alignment with lognormal distribution characteristics in empirical data. This finding challenges existing assumptions about the summation of lognormal variates and provides a more nuanced understanding of approximation techniques.

The research contributes to the field by offering a robust methodological framework for selecting approximation methods. The Wilkinson approach emerged as particularly effective, providing researchers and practitioners with a reliable tool for statistical modeling in complex systems. The method’s performance underscores the importance of rigorous statistical testing and careful method selection, especially in domains where precise distribution estimation is crucial.

The practical implications of this research extend beyond methodological refinement. The findings provide valuable guidance for statistical modeling in finance, engineering, and scientific research, where accurate distribution approximation is essential. Researchers can now approach the summation of lognormal variates with a more informed understanding of the most appropriate approximation techniques.

While the Wilkinson approximation showed the most promising results, the study emphasizes the context-dependent nature of statistical approximation. The selection of an approximation method must consider specific computational constraints, accuracy requirements, and the unique characteristics of the underlying dataset. This nuanced approach highlights the complexity of statistical modelling and the need for careful, context-specific methodological choices.

Future research should focus on expanding the investigation to more diverse datasets, exploring the underlying mathematical mechanisms that contribute to the Wilkinson method’s effectiveness, and developing more sophisticated approximation techniques for correlated lognormal variates. The current study provides a solid foundation for these continued investigations, offering a comprehensive approach to understanding and addressing the challenges of lognormal variate summation.

## Supporting information

S1 Table Data of fatigue life (Company A). (PDF)

S2 Table Data of stock price for six companies. (PDF)

S1 Text Example coding of normal simulation. (PDF)
