# Supplementary material for: On the approximation of sum of lognormal for correlated variates and implementation
Source: PLoS One. 2025 Jun 23;20(6):e0325647. doi: 10.1371/journal.pone.0325647 (PMC12184950; doi:10.1371/journal.pone.0325647)
Supplement: S1 Table — (PDF) [file pone.0325647.s001.PDF]

Data of Fatigue Life (Company A)

No	L10	L50
1	19.2	84.5
2	26.2	74.2
3	11.1	68.1
4	11.8	66.8
5	13.5	79.4
6	5.8	25.7
7	18.3	44.7
8	5.62	73.2
9	15.8	82.7
10	8.7	41.6
11	11.6	160
12	20.6	71.4
13	14.5	88.2
14	12.1	33.1
15	15.1	46.4
16	14	43.6
17	19.3	51.8
18	46.2	110
19	30	88.2
20	21.1	57.4
21	17.3	45.7
22	37.5	118
23	20.3	77.1
24	4.03	42.5
25	8.38	84.7
26	1.79	13.5
27	11.7	45.1
28	4.15	15.8
29	7.23	41
30	22.9	110
31	9.54	31.6
32	6.28	23
33	4.81	21.2
34	4.17	12.8
35	5.42	31.6
36	7.47	49.5
37	4.8	21.3
38	14.8	78.4
39	84.9	460
40	3.4	16.5
41	1.24	3.23
42	241	951
43	3.01	12.6
44	89.1	486
45	15.2	104

46	2.04	10.2
47	51	376
48	5.26	58.8
49	0.883	4.94
50	14.8	57.4

Data of Fatigue Life (Company B)

No	L10	L50
1	6.68	13.4
2	29.8	70
3	16.3	55.1
4	28.5	69.2
5	16.4	49.3
6	10.3	40.1
7	25.7	46.4
8	9.55	39.6
9	17.9	62.1
10	19.9	73.2
11	12.9	50.4
12	19.3	46.2
13	11.1	23.3
14	15.7	43.5
15	11.2	38.1
16	417	809
17	216	709
18	35.6	100
19	12	42.2
20	8.53	46.6
21	6.77	18.9
22	13.5	46.5
23	2.32	8.06
24	36.7	141
25	19	57.2
26	19.5	60.6
27	17	74.4
28	20.9	53.7
29	9.56	40.7
30	5.49	33.3
31	1.39	44
32	9.8	82.7
33	5.19	54.9
34	6.36	17.5
35	3.68	22.1
36	8.34	23.6
37	6.78	36.4
38	9.27	18.4

39	18.2	56.9
40	22.8	56.2
41	3.99	15.6
42	9.07	29.4
43	7.14	28.5
44	12.5	26.4
45	18.8	48.7
46	21.5	53.2
47	17.1	59
48	15.2	87.6
49	3.1	92.3
50	15	47.6
51	17.5	52.8
52	14.4	65.6
53	8.76	22.1
54	12.1	43.3
55	17.2	64.6
56	10.7	34.6
57	10.9	37.6
58	12.7	53.7
59	3.73	43.5
60	16.6	78.3
61	180	275
62	85.2	234
63	57.1	230
64	15.7	55.8
65	27.1	97.8
66	21.7	122
67	13.2	42.3
68	35.8	145
69	12.7	34.7
70	10.1	27.8
71	8.83	34.3
72	16.5	60.3
73	17.9	65.8
74	15.7	63.1
75	10.8	42.1
76	14.2	39.9
77	19	67.8
78	16.3	57.7
79	2.93	18
80	5.69	25.4
81	9.54	39.9
82	12.6	55.7
83	5.1	37.5
84	16	53.7
85	1.98	22.1
86	5.65	28.8

87	12.8	43.6
88	9.84	32.3
89	12.1	43
90	5.48	40.8
91	6.64	25.3
92	13.9	41.9
93	9.02	45.4
94	11	49.2
95	14.5	73.6
96	5.91	37.2
97	18.1	40.5
98	17.1	53.3
99	32.6	61.8
100	24.1	66.2
101	36.1	71.6
102	63.3	104
103	14.4	59
104	15.1	92.9
105	18.8	39.4
106	5.63	34.7
107	7.23	34.5
108	16.7	71.8
109	26.5	90.3
110	8.35	49.1
111	3.79	9.3
112	9.05	36.6
113	2.98	7.35
114	22.5	73.4
115	3.82	31.7
116	6.55	20.8
117	17.5	64.3
118	61.7	152
119	18.6	42.7
120	21.6	66.3
121	11.9	39.1
122	13.9	50.6
123	7.8	33.1
124	3.55	13.9
125	9.4	23.4
126	4.76	22.7
127	3.23	9.86
128	2.62	9.52
129	7.89	39.7
130	4.93	20.4
131	6.26	16.2
132	37.3	103
133	14	38.6
134	30.3	87.6

135	25.7	71.2
136	10.5	60.4
137	10.3	24.1
138	4.56	12.9
139	25.1	274
140	48.8	264
141	7.53	60.7
142	14.9	62.6
143	4.57	43.4
144	3.9	40.7
145	15.5	79.4
146	10.2	43.9
147	4.71	16.9
148	10.1	34.2

Data of Fatigue Life (Company C)

No	L10	L50
1	16.9	64.8
2	211	729
3	74.4	287
4	9.62	40.1
5	11.9	66.3
6	13.8	58
7	2.38	11.3
8	2.38	11.5
9	8.75	62.2
10	25.7	113
11	14	113
12	26.8	65.6
