# Supplementary material for: On the approximation of sum of lognormal for correlated variates and implementation
Source: PLoS One. 2025 Jun 23;20(6):e0325647. doi: 10.1371/journal.pone.0325647 (PMC12184950; doi:10.1371/journal.pone.0325647)
Supplement: S2 Table — (PDF) [file pone.0325647.s002.PDF]

Data of Stock Price for Six Companies

Date	AAPL	AMZN	BRKB	GOOG	META	MSFT
9/5/2023	188.734	137.27	360.47	136.555	299.534	331.065
9/6/2023	181.979	135.36	361.67	135.216	298.556	330.4
9/7/2023	176.656	137.85	361.8	136.045	298.058	327.452
9/8/2023	177.273	138.23	363.15	137.044	297.279	331.78
9/11/2023	178.447	143.1	365.52	137.583	306.929	335.422
9/12/2023	175.402	141.23	367.78	135.915	301.041	329.298
9/13/2023	173.323	144.85	367.82	137.344	304.434	333.556
9/14/2023	174.845	144.72	369.5	138.832	311.081	336.177
9/15/2023	174.119	140.39	367.86	138.143	299.694	327.76
9/18/2023	177.064	139.98	370.43	138.802	301.93	326.608
9/19/2023	178.158	137.63	370.48	138.672	304.444	326.202
9/20/2023	174.597	135.29	366.82	134.437	299.055	318.38
9/21/2023	173.045	129.33	363.28	131.211	295.124	317.149
9/22/2023	173.9	129.12	360.16	131.101	298.467	314.648
9/25/2023	175.184	131.27	361.71	132.02	300.213	315.174
9/26/2023	171.085	125.98	359.42	129.303	298.347	309.815
9/27/2023	169.562	125.98	357.78	131.311	297.129	310.46
9/28/2023	169.821	125.98	357.06	132.979	303.337	311.303
9/29/2023	170.338	127.12	350.3	131.7	299.594	313.398
10/2/2023	172.865	129.46	348.08	135.016	306.191	319.403
10/3/2023	171.522	124.72	343.04	133.148	300.323	311.055
10/4/2023	172.776	127	343.69	136.115	304.953	316.584
10/5/2023	174.02	125.96	345.06	135.835	304.165	316.981
10/6/2023	176.586	127.96	346.34	138.572	314.783	324.822
10/9/2023	178.079	128.26	345.45	139.341	317.707	327.363
10/10/2023	177.482	129.48	348.56	139.042	321.18	325.943
10/11/2023	178.885	131.83	348.43	141.539	327.148	329.943
10/12/2023	179.79	132.33	345.66	140.131	323.495	328.693
10/13/2023	177.939	129.79	345.09	138.422	314.045	325.288
10/16/2023	177.81	132.55	346.23	140.33	320.491	330.162
10/17/2023	176.248	131.47	345.39	140.83	323.336	329.586
10/18/2023	174.945	128.13	340.89	139.122	316.32	327.651
10/19/2023	174.567	128.4	338.66	138.822	312.169	328.852
10/20/2023	172	125.17	335.86	136.585	308.017	324.236
10/23/2023	172.119	126.56	336.84	137.743	313.366	326.867
10/24/2023	172.557	128.56	338.63	139.961	311.909	328.068
10/25/2023	170.229	121.39	336.9	126.526	298.916	338.132
10/26/2023	166.04	119.57	336.16	123.3	287.759	325.447
10/27/2023	167.364	127.74	331.71	123.26	296.122	327.353
10/30/2023	169.423	132.71	337.41	125.607	302.039	334.797
10/31/2023	169.901	133.09	341.33	125.158	300.652	335.591
11/1/2023	173.084	137	343.75	127.425	311.21	343.492
11/2/2023	176.666	138.07	349.02	128.434	310.232	345.725
11/3/2023	175.751	138.6	351.81	130.222	313.955	350.172
11/6/2023	178.318	139.74	346.63	131.301	315.152	353.874

11/7/2023	180.894	142.71	346.17	132.249	318.166	357.844
11/8/2023	181.959	142.08	346.3	133.109	319.124	360.494
11/9/2023	181.481	140.6	348.18	131.54	319.893	358.003
11/10/2023	185.695	143.56	350.56	133.908	328.096	366.916
11/13/2023	184.101	142.59	350.01	133.488	328.515	363.948
11/14/2023	186.731	145.8	354.25	135.276	335.62	367.511
11/15/2023	187.299	143.2	356.79	136.225	332.028	367.661
11/16/2023	188.993	142.83	359.86	138.542	333.505	374.125
11/17/2023	188.973	145.18	358.93	136.784	334.353	367.84
11/20/2023	190.726	146.13	361.33	137.763	339.273	375.388
11/21/2023	189.919	143.9	361	138.462	336.289	371.042
11/22/2023	190.587	146.71	361.8	139.861	340.79	375.796
11/24/2023	189.252	146.74	362.68	138.063	337.536	375.378
11/27/2023	189.073	147.73	361.34	137.893	334.014	376.552
11/28/2023	189.68	147.03	360.05	138.462	338.295	380.62
11/29/2023	188.654	146.32	358.69	136.245	331.519	376.791
11/30/2023	189.232	146.09	360	133.768	326.479	376.85
12/1/2023	190.517	147.03	357.07	133.168	324.154	372.474
12/4/2023	188.714	144.84	356.66	130.482	319.364	367.134
12/5/2023	192.689	146.88	355.03	132.24	317.637	370.495
12/6/2023	191.593	144.52	352.38	131.281	316.799	366.795
12/7/2023	193.536	146.88	352.7	138.293	325.92	368.934
12/8/2023	194.97	147.42	353.08	136.485	332.068	372.196
12/11/2023	192.45	145.89	357.06	134.547	324.613	369.282
12/12/2023	193.974	147.48	360.02	133.488	333.535	372.345
12/13/2023	197.212	148.84	362.03	133.818	334.054	372.335
12/14/2023	197.361	147.42	358.12	133.049	332.487	363.941
12/15/2023	196.823	149.97	356.5	133.688	334.233	368.715
12/18/2023	195.149	154.07	359.65	137.034	343.913	370.624
12/19/2023	196.196	153.79	361.8	137.943	349.642	371.231
12/20/2023	194.093	152.12	355.35	139.501	348.564	368.605
12/21/2023	193.944	153.84	356.14	141.639	353.364	371.51
12/22/2023	192.868	153.42	356.47	142.558	352.665	372.544
12/26/2023	192.32	153.41	356.83	142.658	354.102	372.624
12/27/2023	192.42	153.34	356.95	141.279	357.096	372.037
12/28/2023	192.848	153.38	357.57	141.119	357.585	373.24
12/29/2023	191.802	151.94	356.66	140.77	353.234	373.996
1/2/2024	184.938	149.93	362.46	139.401	345.58	368.854
1/3/2024	183.553	148.47	366.75	140.2	343.764	368.586
1/4/2024	181.222	144.57	363.68	137.883	346.408	365.94
1/5/2024	180.495	145.24	365.59	137.234	351.228	365.751
1/8/2024	184.859	149.1	368.18	140.37	357.925	372.653
1/9/2024	184.44	151.37	366.9	142.398	356.697	373.747
1/10/2024	185.486	153.73	367.92	143.637	369.71	380.689
1/11/2024	184.888	155.18	363.34	143.507	368.912	382.539
1/12/2024	185.217	154.62	363.62	144.076	373.722	386.358
1/16/2024	182.936	153.16	361.1	143.916	366.706	388.149
1/17/2024	181.989	151.71	359.29	142.728	367.615	387.353

1/18/2024	187.917	153.5	362.38	144.825	375.359	391.729
1/19/2024	190.836	155.34	366.99	147.802	382.664	396.503
1/22/2024	193.157	154.78	368.06	147.542	380.997	394.355
1/23/2024	194.442	156.02	372.14	148.511	384.41	396.732
1/24/2024	193.765	156.87	376.59	150.179	389.899	400.372
1/25/2024	193.436	157.75	380.85	153.465	392.374	402.669
1/26/2024	191.693	159.12	385.4	153.615	393.332	401.734
1/29/2024	191.005	161.26	383.18	154.664	400.198	407.493
1/30/2024	187.329	159	387.15	152.876	399.24	406.369
1/31/2024	183.703	155.2	383.74	141.639	389.34	395.419
2/1/2024	186.154	159.28	386.44	142.548	393.97	401.585
2/2/2024	185.147	171.81	390.75	143.377	474.016	408.985
2/5/2024	186.971	170.31	390.76	144.765	458.468	403.445
2/6/2024	188.584	169.15	393.74	145.245	453.788	403.286
2/7/2024	188.694	170.53	397.66	146.513	468.627	411.799
2/8/2024	187.608	169.84	397.49	147.053	469.036	411.859
2/9/2024	188.376	174.45	398.36	150.049	467.15	418.264
2/12/2024	186.68	172.34	397.71	148.561	467.938	413.003
2/13/2024	184.576	168.64	394.8	146.204	459.176	404.111
2/14/2024	183.688	170.98	398.68	146.973	472.309	408.017
2/15/2024	183.399	169.8	403.45	143.776	483.037	405.098
2/16/2024	181.853	169.51	405.99	141.599	472.349	402.607
2/20/2024	181.104	167.08	407.15	142.038	470.783	401.341
2/21/2024	181.863	168.59	409.25	143.676	467.566	400.734
2/22/2024	183.907	174.58	415.16	145.155	485.648	410.17
2/23/2024	182.062	174.99	417.22	145.125	483.55	408.864
2/26/2024	180.705	174.73	409.14	138.592	481.262	406.074
2/27/2024	182.172	173.54	408.91	139.941	486.567	406.015
2/28/2024	180.965	173.16	412.14	137.274	483.54	406.254
2/29/2024	180.296	176.76	409.4	139.621	489.644	412.152
3/1/2024	179.209	178.22	407.11	137.923	501.802	414.006
3/4/2024	174.661	177.58	403.39	134.047	497.696	413.428
3/5/2024	169.693	174.12	400.74	133.628	489.734	401.202
3/6/2024	168.696	173.51	403.96	132.409	495.598	400.644
3/7/2024	168.576	176.82	402.39	135.086	511.682	407.669
3/8/2024	170.302	175.35	403.15	136.135	505.448	404.759
3/11/2024	172.317	171.96	404.76	138.782	483.11	403.065
3/12/2024	172.795	175.39	404.98	139.461	499.254	413.786
3/13/2024	170.701	176.56	408.13	140.61	495.078	413.607
3/14/2024	172.566	178.75	406.73	144.176	491.342	423.691
3/15/2024	172.187	174.42	408.13	142.008	483.62	414.922
3/18/2024	173.284	174.48	408.41	148.311	496.487	415.819
3/19/2024	175.638	175.9	411.76	147.752	495.748	419.894
3/20/2024	178.222	178.15	416.11	149.51	505.019	423.701
3/21/2024	170.94	178.15	413.78	148.571	507.256	427.826
3/22/2024	171.848	178.87	411.6	151.597	509.075	427.198
3/25/2024	170.421	179.71	409.92	150.978	502.521	421.339
3/26/2024	169.284	178.3	411.57	151.528	495.398	420.134

3/27/2024	172.875	179.83	416.93	151.767	493.37	419.914
3/28/2024	171.05	180.38	420.52	152.087	485.098	419.207
4/1/2024	169.603	180.97	420.2	156.322	490.863	423.043
4/2/2024	168.416	180.69	418.92	155.693	496.877	419.924
4/3/2024	169.224	182.41	420.24	156.192	506.237	418.938
4/4/2024	168.396	180	415.32	151.767	510.413	416.377
4/5/2024	169.154	185.07	418.62	153.765	526.817	423.99
4/8/2024	168.027	185.19	415.97	155.963	518.735	423.063
4/9/2024	169.244	185.67	414.69	157.96	516.387	424.747
4/10/2024	167.359	185.95	409.11	157.481	519.314	421.738
4/11/2024	174.601	189.05	407.61	160.607	522.641	426.391
4/12/2024	176.107	186.13	403.26	159.009	511.392	420.383
4/15/2024	172.257	183.62	400.26	156.152	499.734	412.152
4/16/2024	168.955	183.32	396.92	155.823	499.264	413.089
4/17/2024	167.578	181.28	397.74	156.702	493.68	410.359
4/18/2024	166.621	179.22	399.89	157.281	501.302	402.816
4/19/2024	164.586	174.63	405.08	155.543	480.593	397.685
4/22/2024	165.424	177.23	408.78	157.77	481.252	399.518
4/23/2024	166.481	179.54	408.74	159.738	495.608	406.104
4/24/2024	168.596	176.59	405.95	160.917	493.01	407.589
4/25/2024	169.464	173.67	404.91	157.77	440.942	397.605
4/26/2024	168.875	179.62	402.1	173.493	442.85	404.859
4/29/2024	173.065	180.96	400.96	167.709	432.191	400.803
4/30/2024	169.903	175	396.73	164.453	429.743	387.93
5/1/2024	168.875	179	398.58	165.382	438.754	393.52
5/2/2024	172.596	184.72	400.6	168.269	441.242	396.409
5/3/2024	182.92	186.21	400.87	168.798	451.512	405.197
5/6/2024	181.254	188.7	404.92	169.637	465.218	412.053
5/7/2024	181.942	188.76	406.14	172.783	467.776	407.868
5/8/2024	182.281	188	406.37	170.965	472.131	409.064
5/9/2024	184.107	189.5	408.82	171.385	474.948	410.837
5/10/2024	182.838	187.48	412.05	170.096	475.728	413.248
5/13/2024	186.065	186.57	411.22	170.706	467.546	412.232
5/14/2024	187.213	187.07	410.24	171.735	471.382	415.062
5/15/2024	189.501	185.99	412.76	173.682	481.062	422.319
5/16/2024	189.621	183.63	413.12	175.231	472.761	420.233
5/17/2024	189.65	184.7	416.94	177.088	471.442	419.454
5/20/2024	190.819	183.54	413	178.257	468.375	424.575
5/21/2024	192.128	183.15	414.37	179.336	464.169	428.268
5/22/2024	190.679	183.13	413.99	177.798	467.316	429.745
5/23/2024	186.664	181.05	405.88	174.861	465.318	426.232
5/24/2024	189.76	180.75	407.41	176.13	477.746	429.386
5/28/2024	189.77	182.15	403.9	177.818	479.444	429.546
5/29/2024	190.07	182.02	404.09	177.198	473.889	428.398
5/30/2024	191.069	179.32	408.61	173.363	466.587	413.924
5/31/2024	192.028	176.44	414.4	173.762	466.367	414.383
6/3/2024	193.806	178.34	414.79	174.222	477.016	412.776
6/4/2024	194.125	179.34	409.53	174.931	476.517	415.321

6/5/2024	195.644	181.28	409.85	176.869	494.569	423.247
6/6/2024	194.255	185	411.08	178.147	493.27	423.756
6/7/2024	196.662	184.3	413.72	175.75	492.471	423.087
6/10/2024	192.897	187.06	410.81	176.63	502.102	427.1
6/11/2024	206.91	187.23	408.5	178.19	506.967	431.902
6/12/2024	212.824	186.89	408.77	179.56	508.335	440.266
6/13/2024	213.992	183.83	405.92	176.74	503.6	440.786
6/14/2024	212.244	183.66	405.54	178.37	504.16	441.774
6/17/2024	216.419	184.06	407.32	178.78	506.63	447.563
6/18/2024	214.042	182.81	407.95	176.45	499.49	445.537
6/20/2024	209.438	186.1	409.15	177.71	501.7	444.898
6/21/2024	207.25	189.08	409.62	180.26	494.78	448.971
6/24/2024	207.899	185.57	413.98	180.79	498.91	446.865
6/25/2024	208.828	186.34	410.97	185.58	510.6	450.139
6/26/2024	213.003	193.61	410.26	185.37	513.12	451.346
6/27/2024	213.852	197.85	407.95	186.86	519.56	452.035
6/28/2024	210.376	193.25	406.8	183.42	504.22	446.146
7/1/2024	216.499	197.2	405.19	184.49	504.68	455.908
7/2/2024	220.015	200	407.1	186.61	509.5	458.454
7/3/2024	221.294	197.59	405.77	187.39	509.96	459.941
7/5/2024	226.078	200	411.17	191.96	539.91	466.719
7/8/2024	227.557	199.29	409.08	190.48	529.32	465.401
7/9/2024	228.416	199.34	410.52	190.44	530	458.713
7/10/2024	232.711	199.79	413.79	192.66	534.69	465.411
7/11/2024	227.307	195.05	418.78	187.3	512.7	453.882
7/12/2024	230.273	194.49	424.44	186.78	498.87	452.734
7/15/2024	234.129	192.72	434.42	188.19	496.16	453.143
7/16/2024	234.549	193.02	438.9	185.5	489.79	448.711
7/17/2024	228.615	187.93	445.61	182.62	461.99	442.722
7/18/2024	223.921	183.75	441.82	179.22	475.85	439.578
7/19/2024	224.051	183.13	434.47	179.39	476.79	436.324
7/22/2024	223.701	182.55	435.98	183.35	487.4	442.143
7/23/2024	224.75	186.41	434.01	183.6	488.69	444.05
7/24/2024	218.287	180.83	432.8	174.37	461.27	428.128
7/25/2024	217.239	179.85	433.29	169.16	453.41	417.647
7/26/2024	217.708	182.5	437.66	168.68	465.7	424.505
7/29/2024	217.988	183.2	438.31	171.13	465.71	425.962
7/30/2024	218.547	181.71	441.26	171.86	463.19	422.159
7/31/2024	221.823	186.98	438.5	173.15	474.83	417.597
8/1/2024	218.108	184.07	431.81	172.45	497.74	416.36
8/2/2024	219.606	167.9	428.36	168.4	488.14	407.755
8/5/2024	209.028	161.02	413.72	160.64	475.73	394.439
8/6/2024	206.99	161.93	422.14	160.54	494.09	398.891
8/7/2024	209.577	162.77	427.02	160.75	488.92	397.713
8/8/2024	213.063	165.8	431.54	163.84	509.63	401.965
8/9/2024	215.99	166.94	431.67	165.39	517.77	405.289
8/12/2024	217.53	166.8	430.39	163.95	515.95	406.078
8/13/2024	221.27	170.23	432.41	165.93	528.54	413.265

8/14/2024	221.72	170.1	438.47	162.03	526.76	416.11
8/15/2024	224.72	177.59	440.84	163.17	537.33	421.03
8/16/2024	226.05	177.06	444.51	164.74	527.42	418.47
8/19/2024	225.89	178.22	448.77	168.4	529.28	421.53
8/20/2024	226.51	178.88	448.36	168.96	526.73	424.8
8/21/2024	226.4	180.11	446.6	167.63	535.16	424.14
8/22/2024	224.53	176.13	449.03	165.49	531.93	415.55
8/23/2024	226.84	177.04	453.38	167.43	528	416.79
8/26/2024	227.18	175.5	454.49	167.93	521.12	413.49
8/27/2024	228.03	173.12	460.63	166.38	519.1	413.84
8/28/2024	226.49	170.8	464.59	164.5	516.78	410.6
8/29/2024	229.79	172.12	468.37	163.4	518.22	413.12
8/30/2024	229	178.5	475.92	165.11	521.31	417.14
