# Supplementary material for: On the approximation of sum of lognormal for correlated variates and implementation
Source: PLoS One. 2025 Jun 23;20(6):e0325647. doi: 10.1371/journal.pone.0325647 (PMC12184950; doi:10.1371/journal.pone.0325647)
Supplement: S1 Text — (PDF) [file pone.0325647.s003.PDF]

Example Coding of Normal Simulation

```
library(LaplacesDemon)
library(moments)
library(ADGofTest)
library(MASS)
#rho=0.9

CorrNorm <- function(n=30, rho = 0.9)
  {
   X1 = matrix(rlnorm(30,0,4),ncol=30); X2 = matrix(rlnorm(30,20,12),ncol=30)
   W1 = cbind(X1, rho*X1+sqrt(1-rho^2)*X2)
   return(W1)
  }

x1<-Z[1,c(1:30)]
x2<-Z[1,c(31:60)]
w1<-matrix(x1+x2,ncol=30)
```

Wilkinson Approximation

```
mle1<-fitdistr(w1,"lognormal")
minW1<-  21.064942
sdW1<-   11.459327
pv1<-apply(w1,2,function(x)ad.test(x,plnorm,minW1,sdW1)$p.value)
alpha1<-sum(pv1<0.05)
```

Schwartz dan Yeh Approximation

```
minlnW1<-log(abs(minW1))
sdlnW1<-sqrt(((log((minW1)^2))^2)-(minlnW1)^2)
pv1<-apply(w1,2,function(x)ad.test(x,plnorm,minlnW1,sdlnW1)$p.value)
alpha1<-sum(pv1<0.05)
```

Inverse Approximation

```
a<-matrix(data=c(1,0.5,2,2),nrow=2,ncol=2,byrow=TRUE)
b<-
matrix(data=c(log(abs(minW1)),log(((sdW1)^2+(minW1)^2))),nrow=2,ncol=1,byrow=FALSE)
round(solve(a,b),3)
m<-   2.918
```

```
sdev<-sqrt(0.259)
m1<-exp(-m+((sdev)^2/2))
m2<-exp((-2*m)+(2*(sdev^2)))
minlnW1<-(0.5*log(m2))-(2*log(m1))
sdlnW1<-sqrt(log(m2)-(2*log(m1)))
pv1<-apply(w1,2,function(x)ad.test(x,plnorm,minlnW1,sdlnW1)$p.value)
alpha1<-sum(pv1<0.05)
```
